# The effects of high-intensity interval training at the anaerobic and psychomotor fatigue thresholds on physiological parameters in young soccer players: a prospective study

**DOI:** 10.3389/fphys.2023.1221121

**Published:** 2023-09-07

**Authors:** Paweł Chmura, Jan Chmura, Wiktor Chodor, Adrian Drożdżowski, Andrzej Rokita, Marek Konefał

**Affiliations:** ^1^ Department of Team Games, Wroclaw University of Health and Sport Sciences, Wroclaw, Poland; ^2^ Latvian Academy of Sport Education, Rīga LV, Latvia; ^3^ Department of Human Motor Skills, Wroclaw University of Health and Sport Sciences, Wroclaw, Poland

**Keywords:** football, heart rate, global positioning system, maximal oxygen consumption, repeated small-sided games

## Abstract

This study aimed to investigate the effects of a 4-week specific high-intensity interval training (HIIT) program performed between the anaerobic threshold (ANT) and the psychomotor fatigue threshold (PFT) on physiological parameters in 14 professional soccer players at the under-17 level. The first and second stages of the research protocol included a treadmill running exercise with increasing load and six 3-min four-versus-four games of soccer with a 3-min break between games. Players then participated in a training microcycle involving three specific HIIT exercises twice per week for 4 weeks, after which they repeated stages one and two, followed by an assessment of changes. The measurement of lactate (LA) determined ANT, whereas the choice reaction time (CRT) indicated PFT among other selected physiological parameters. The repeated-measure analysis of variance (ANOVA) compared mean values for the examined variables using Bonferroni *post hoc* test. It demonstrated significantly increased maximal oxygen consumption (VO_2_ max) from 45.9 ± 3.0 to 48.7 ± 2.6 at the ANT and from 49.1 ± 3.4 to 52.0 ± 3.6 on the PFT after 4 weeks of training. A significant increase in the running speed (RS) at both thresholds and heart rate (HR) at the ANT (*p* ≤ 0.05) was also recorded. Moreover, the players exceeded their intensity of effort during ANT while playing four-versus-four soccer matches, but they did not reach intensity during PFT. In conclusion, the findings of the study demonstrated that both thresholds shifted toward higher loads and the proposed specific HIIT effectively increased the exercise capacity of soccer players.

## Introduction

High-intensity interval training (HIIT) is very popular in modern soccer (Buchheit and Laursen, 2019), with one of the benefits being its time efficiency in improving performance and physiological parameters (Costigan et al., 2015; García-Hermoso et al., 2016). Furthermore, HIIT provides sufficient stimulus for anaerobic parameter improvements, such as the ability to perform straight-line sprints, repeated sprints, and jumps (Sperlich et al., 2011; Tønnessen et al., 2011; Ferrete et al., 2014). Systematic HIIT training also had a substantial positive effect on cardiovascular and respiratory fitness and increased maximal oxygen consumption (VO_2_ max) (Eddolls et al., 2017; Thivel et al., 2019). HIIT involves various interval protocols with varying exercise durations and recovery intervals (Kunz et al., 2019), and studies that have tested the short-interval or long-interval HIIT demonstrated moderate to high benefits (Helgerud et al., 2001; Bravo et al., 2008; Ouerghi et al., 2014).

Effective HIIT requires the identification of the individual intensity that optimally stimulates exercise capacity development. Determining the minimum intensity with which exercises should be performed is often based on an individual anaerobic threshold (ANT), which is usually 75%–85% of the maximum heart rate (HR max), although the maximum load value varies in different protocols and can reach 90%–95% of HR max (Los Arcos et al., 2015; Kunz et al., 2019). However, another threshold observed above the ANT, the psychomotor fatigue threshold (PFT), is crucial in soccer. PFT determines the individual load at which the central nervous system (CNS) reaches its highest operational efficiency and fatigue tolerance (Chmura and Nazar, 2010) and often occurs between 88% and 92% of HR max (Chmura and Nazar, 2010). As such, intensity above the ANT benefits the development of motor skills, while intensities closer to PFT improve cognitive processes (Konefał et al., 2022). To date, there are few studies in this area (Chmura et al., 1998; Andrzejewski et al., 2011). For example, (Irandoust and Taheri, 2017; Paryab et al., 2021) indicated the positive effect of melatonin supplementation on improving psychomotor and physical performance. Therefore, it seems reasonable to propose a modified HIIT protocol that accounts for the level of effort between the two thresholds.

Training exercises performed with the ball are most valued in soccer, meaning that small-sided games (SSGs) are very popular (Moreira et al., 2016; Clemente et al., 2021) and can increase exercise motivation compared to traditional fitness training because they are a soccer-specific method. Furthermore, SSGs are considered to be more time efficient, as physical performance, technical skills, and tactical awareness can develop simultaneously (Aguiar et al., 2012). Additionally, they provide many practical benefits for all ages and sport levels (Clemente et al., 2021). Movement requirements, physiological changes, technical requirements, decision-making under time pressure, opponent, and fatigue are similar to the match effort (Hill-Haas et al., 2011; Aşçı, 2016). Like HIIT protocols, different sizes and formats of SSGs can elicit various physiological and cognitive responses and generate different exercise intensities (Brandes et al., 2012). Most studies report an increase in HR as the playing area increases and the number of players decreases (Hill-Haas et al., 2011; Randers et al., 2014; Arslan et al., 2017). A proven training exercise is the four-versus-four games (Konefał et al., 2023), which generate high-intensity exercise and may increase VO_2_ max (Little, 2009).

Nowadays, due to the fact that the schedule is often congested, soccer players play more games per season, and the time available for training is decreasing. Hence, coaches and training staffs are constantly looking for time-efficient and effective exercises to boost players’ performance. Traditional HIIT involves repetitive efforts at constant stimulus intensity (Buchheit and Laursen, 2019), although using HIIT training with variable stimulus intensity between ANT and PFT is interesting from a cognitive point of view. Indeed, exercise at an intensity close to PFT will result in the highest CNS efficiency and stimulate the development of cognitive processes while maintaining the widely known benefits of high-intensity repeated effort (Chmura and Nazar, 2010). Furthermore, it is interesting from a practical point of view to determine if playing an SSG of soccer (four versus four on a 25 m by 35 m pitch) elicits any intensity between the ANT and PFT.

Therefore, this study aimed to assess the effects of specific HIIT training performed between ANT and PFT on the physiological parameters of young soccer players. In addition, this study aimed to determine if the participants could exceed the ANT and achieve PFT intensity during a four-versus-four soccer match on a 25 m by 35 m pitch. In this regard, it was assumed that a 4-week HIIT program would significantly increase the physiological parameters and that young soccer players performing repeated SSGs would at least exceed their ANT each time.

## Materials and methods

### Experimental approach

**FIGURE 1:**
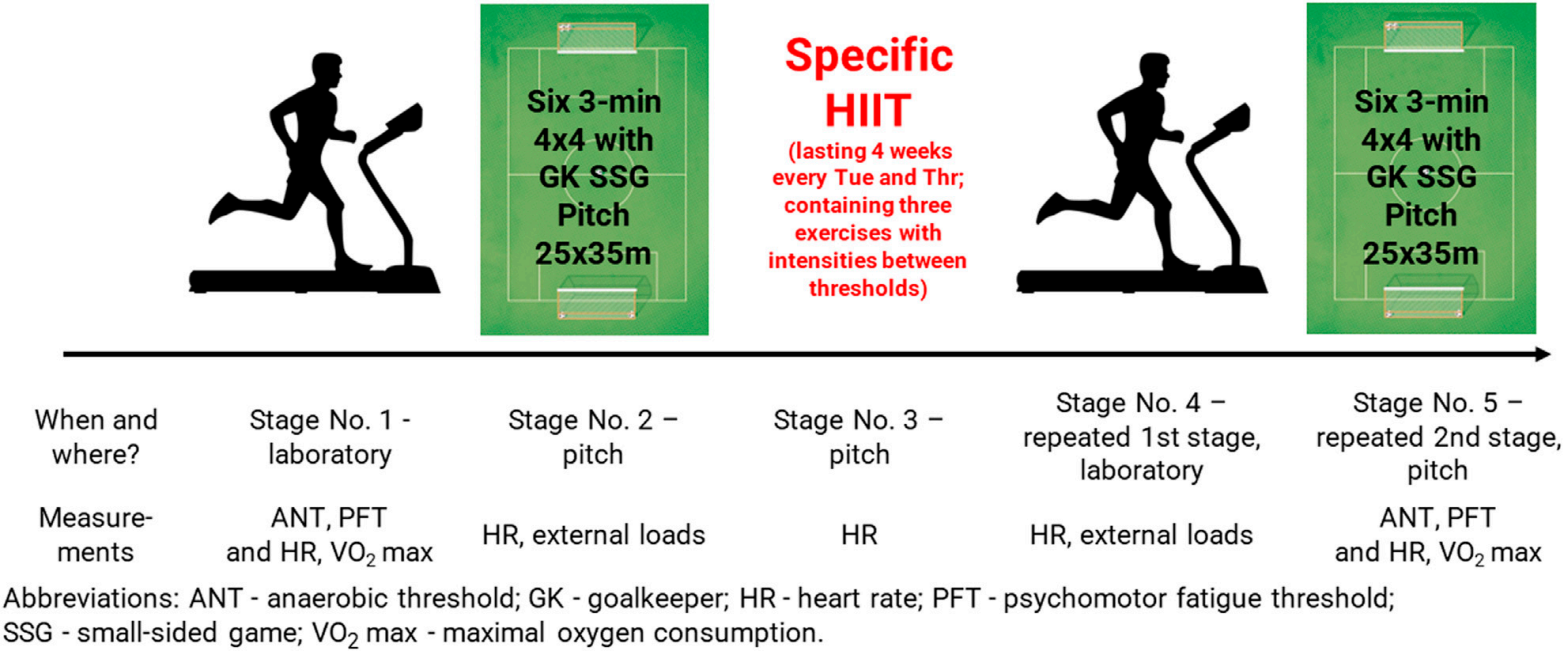
Study design.

The research comprised five stages and commenced with a laboratory-based treadmill running exercise test that used an increasing load (Figure 1). The next stage involved participants playing six 3-min soccer matches (four versus four), with a 3-min break between games. During the third stage, participants performed a specific HIIT program involving three exercises twice a week for 4 weeks. The fourth and fifth stages included the assessment of changes in physiological variables during the repeated performances of stages one and two. The physiological variables assessed included lactate (LA) for determining the ANT, choice reaction time (CRT) to determine the PFT, and other selected parameters.

### Participants and eligibility

Sixteen soccer players competing at the under-17 level for a professional sports club in the second Polish league participated in the study. During the study, two players were unable to complete the experiment due to muscle or tendon injury and were excluded. Hence, fourteen players were included in the analysis. The physical activity of all outfield players was analyzed, though goalkeepers were excluded from the analysis due to the specificity of the effort. Inclusion criteria included a minimum of 8 years of training experience, participation in training at least four times per week before the start of the study, complete laboratory results, and no recent surgery or injury. All players were instructed not to consume alcohol for at least 24 h before testing, to stay hydrated, and follow a normal varied diet.

All participants were briefed with a detailed explanation of the proposed study and its requirements and provided written informed consent directly or through a parent or guardian. They were also informed of the potential risks and could withdraw at any time without any consequences. The study followed guidelines approved by the local ethics committee (12/2021) and the requirements set out in the Declaration of Helsinki, and all health and safety procedures were maintained.

### Procedures and measurements

The research was carried out by qualified research personnel at the beginning of the pre-season period (from 26 July 2021 to 30 September 2021) and comprised five stages.

Stage one involved a laboratory-based treadmill (Cybex 790T) running exercise tests with increasing loads. Each subject started the test at a speed of 8 km/h, which was increased by 2 km/h every 3 minutes until they could no longer continue.

Immediately before the test and after each load, blood was collected from the fingertip to measure LA concentration, which was used to determine ANT (Konefał et al., 2022). In the last minute of each 3-min load, a CRT psychomotor test was carried out using a reaction meter APR (UNI—PAR, Lubin, Poland). The test determined the PFT using 25 audio–visual stimuli, a detailed description of which was previously published (Chmura et al., 2020). In addition, HR was recorded in beats per minute (bpm) using the Polar RS400, and the Cortex Metamax 3B mobile ergospirometer measured VO_2_ (ml/kg/min).

Stage two comprised six 3-min soccer matches (four *versus* four) that included goalkeepers, with a 3-min break between games. The games were played on a 25 (m) by 35 (m) field as described previously (Konefał et al., 2023).

Before testing, players performed a standard 20-min increasing intensity warm-up that included running, stretching, ball exercises, and repeated starts, accelerations, and decelerations. In order to maintain the intensity of player efforts during soccer matches, the coach would throw another ball into the field when the ball went out of bounds. In addition to that the coaching staff used verbal motivation.

External loads were determined using a global positioning system (GPS) with sampling at 10 Hz and triaxial accelerometer sampling at 100 Hz using the Vector S7 (Catapult Sports, Melbourne, Australia) (Beenham et al., 2017). Data were collected after each session using Catapult Sports’ proprietary software (OpenField) (Pop et al., 2022).

The metrics derived from each of the devices included HR max (bpm), total distance covered (TDC) (m), maximal velocity (V max) (km**·**h^−1^), mean velocity (V mean) (km**·**h^−1^), acceleration (Acc) (number), and deceleration (Dec) (number).

Stage three of the experiment used a training microcycle program of three specific HIIT exercises twice a week on Tuesdays and Thursdays (Table 1). The total experiment duration was 4 weeks, which equates to eight training sessions. In addition, the players followed a standard training program (Table 2). During specific HIIT, each player used a Polar RS400 HR monitor that encompassed a watch for real-time exercise intensity control.

**TABLE 1:** Structure of the weekly training microcycle.

	MD+1	MD-5	MD-4	MD-3	MD-2	MD-1	MD
Training content	Day off	Low/moderate intensity, recovery and skill, and upper body strength	High intensity, SSG, sprint, and plyometric	Moderate intensity and MSG or LSG	High intensity, SSG, and tactical focus	Low/moderate intensity, tactical focus, and reaction speed	Match effort
			**Additional specific HIIT**		**Additional specific HIIT**		

Abbreviations: HIIT, high-intensity interval training; LSG, large-sided games; MD, match day; MSG, medium-sided games; SSG, small-sided games.

**TABLE 2:** Differences in physiological and kinematic parameters before and after the specific high-intensity interval training program (mean ± standard deviation).

Variable	Before	After	F (*p*-value)
**VO**_**2ANT**_ **(ml·kg** ^**-1**^ **·min** ^**-1**^ **)**	45.89 ± 3.00	48.73 ± 2.60	**11.665 (0.005)**
**HR**_**ANT**_ **(bpm)**	176.00 ± 3.42	178.98 ± 4.54	**23.40 (0.001)**
**LA**_**ANT**_ **(au)**	3.10 ± 0.13	3.79 ± 0.21	**101.06 (0.001)**
**V**_**ANT**_ **(km·h** ^**−1**^ **)**	13.21 ± 0.97	14.50 ± 0.94	**43.875 (0.001)**
**VO**_**2PFT**_ **(ml·kg** ^**-1**^ **·min** ^**−1**^ **)**	49.14 ± 3.41	52.03 ± 3.55	**16.833 (0.001)**
**HR**_**PFT**_ **(bpm)**	180.43 ± 5.56	182.07 ± 4.46	1.77 (0.206)
**LA**_**PFT**_ **(au)**	4.22 ± 0.48	5.23 ± 0.66	**85.658 (0.001)**
**V**_**PFT**_ **(km·h** ^**−1**^ **)**	14.29 ± 0.91	15.57 ± 0.94	**43.875 (0.001)**

Abbreviations: VO_2ANT_, maximal oxygen consumption at the anaerobic threshold; HR_ANT_, heart rate at the anaerobic threshold; LA_ANT_, lactate concentration at the anaerobic threshold; V_ANT_, velocity at the anaerobic threshold; VO_2PFT_, maximal oxygen consumption at the psychomotor fatigue threshold; HR_PFT_, heart rate at the psychomotor fatigue threshold; LA_PFT_, lactate concentration at the psychomotor fatigue threshold; V_PFT_, velocity at the psychomotor fatigue threshold. The bold value indicates the statistically significant values. Their exact *p*-value is given in parentheses.

Stage four assessed changes in physiological parameters caused by the applied training by repeating stage one. Meanwhile, stage five measured changes in external loads by repeating stage two.

### Specific high-intensity interval training

**FIGURE 2:**
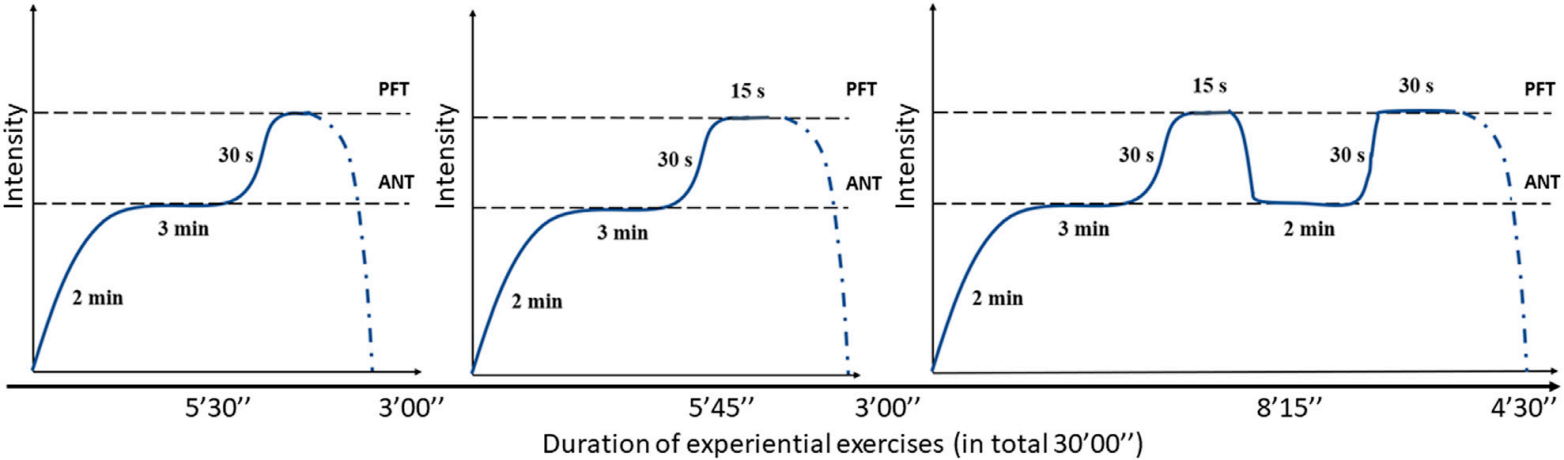
Intensity pattern of three specific high-intensity interval training exercises.

Exercise 1: run with increasing intensity until one reaches the ANT (2 minutes), maintain the running intensity at the ANT for 3 minutes, and then accelerate for 30 seconds until one reaches the PFT intensity. The total duration was 5 minutes and 30 seconds, followed by a 3-min jog (Figure 2).

Exercise 2: run with increasing intensity until one reaches the ANT (2 minutes) and continue running with ANT intensity for 3 minutes, accelerate for 30 seconds until one reaches the PFT, and then run at the PFT for 15 seconds. The total duration was 5 minutes and 45 seconds, followed by a 3-min jog.

Exercise 3: run with increasing intensity until one reaches the ANT (2 minutes) and continue running at the ANT for 3 minutes, then accelerate for 30 seconds until one reaches the PFT, and then run for 15 seconds. Reduce the running speed toward the ANT, maintain this speed for 2 minutes, and then increase the running speed for 30 seconds until one reaches the PFT and continue running for 30 seconds. The total activity duration was 8 minutes and 15 seconds, followed by a jog that lasted for 4 minutes and 30 seconds. The specific HIIT workout duration was 30 minutes.

### Statistical analysis

All statistical analyses employed Statistica version 13.3 software (Dell Inc., OK, United States). The Shapiro–Wilk test verified the normality of data distribution, and arithmetic means and standard deviations were calculated. A repeated-measure analysis of variance (ANOVA) compared mean values for the examined variables (Park et al., 2009) with a Bonferroni *post hoc* test used to assess differences between means. The level of statistical significance was set at *p* ≤ 0.05.

## Results

Fourteen soccer players participated in the study. The mean age of participants was 17.3 ± 0.4 years, their stature was 1.76 ± 0.05 m, and their body mass was 69.27 ± 5.43 kg.

The statistical analysis of physiological and kinematic variables before and after the specific HIIT revealed effects on VO_2ANT_ (F = 11.665 (1); *p* = 0.005), HR_ANT_ (F = 23.400 (1); *p* = 0.001), LA_ANT_ (F = 101.060 (1); *p* = 0.001), V_ANT_ (F = 43.875 (1); *p* = 0.001), VO_2PFT_ (F = 16.833 (1); *p* = 0.001), LA_PFT_ (F = 85.658 (1); *p* = 0.001), and V_PFT_ (F = 43.875 (1); *p* = 0.001). However, there was no significant effect on HR_PFT_ (F = 1.770 (1); *p* = 0.206) (Table 2).

The statistical analysis of the physiological and kinematic variables during the repeated SSGs revealed effects on HR max (F = 4.930 (5); *p* = 0.001), V mean (F = 3.307 (5); *p* = 0.007), and Dec (F = 3.860 (5); *p* = 0.003). There was no significant effect on TDC (F = 1.661 (5); *p* = 0.148), V max (F = 2.570 (5); *p* = 0.056), or Acc (F = 1.825 (5); *p* = 0.112) (Table 3).

**TABLE 3:** Differences in kinematic variables over six 3-min small-sided games (mean ± standard deviation).

Variable	Game						F (*p*-value)	SSD (*p* ≤ 0.05)
	1	2	3	4	5	6		
**HR max (bpm)**	179.75 ± 7.62	179.61 ± 7.69	176.68 ± 8.34	177.68 ± 7.12	178.32 ± 7.62	172.75 ± 13.22	**4.93 (0.001)**	1,2,4,5 > 6
**TDC (m)**	366.38 ± 32.87	376.80 ± 40.42	360.21 ± 39.01	367.29 ± 34.06	365.46 ± 44.56	362.95 ± 40.60	1.661 (0.148)	—
**V max (km·h** ^**−1**^ **)**	19.36 ± 1.72	20.28 ± 1.92	20.26 ± 1.65	20.32 ± 2.02	19.42 ± 1.43	20.52 ± 1.89	2.57 (0.056)	—
**V mean (km·h** ^**−1**^ **)**	7.25 ± 0.64	7.22 ± 0.70	6.93 ± 0.74	7.10 ± 0.61	7.03 ± 0.78	6.89 ± 0.84	**3.307 (0.007)**	1 > 6
**Acc (number)**	22.61 ± 3.55	21.93 ± 4.83	21.71 ± 4.81	20.54 ± 4.40	21.75 ± 3.72	20.71 ± 4.37	1.825 (0.112)	—
**Dec (number)**	22.43 ± 3.78	23.18 ± 4.34	22.36 ± 5.17	21.61 ± 3.73	22.61 ± 4.58	20.11 ± 4.34	**3.860 (0.003)**	1,2,5 > 6

Abbreviations: Acc, acceleration; Dec, deceleration; HR max, maximal heart rate; TDC, total distance covered; V max, maximal velocity; V mean, mean velocity. The bold value indicates the statistically significant values. Their exact *p*-value is given in parentheses.

## Discussion

This study aimed to assess the impact of specific HIIT performed between ANT and PFT on the physiological parameters of young soccer players. Many recently published studies indicated the high effectiveness of HIIT in sports (Fernandez-Fernandez et al., 2017; Monks et al., 2017), which is reflected in the increased levels of physiological and kinematic parameters (Engel et al., 2018). In practice, such increases shift the ANT toward higher loads (Little, 2009; Kunz et al., 2019) and are more frequently determined by measuring the LA concentration (Hill-Haas et al., 2011; Stanula et al., 2013). The major findings of this study showed that the use of the 4-week physical training, comprising three exercises of a specific intensity above the ANT, is sufficient to significantly increase the physical performance of players.

This finding indicates that the proposed specific HIIT, with intensity between anaerobic and psychomotor fatigue thresholds, caused a shift in the ANT toward higher loads. Indeed, HIIT significantly increased VO_2_ by 6.2%, HR by 1.7%, and running speed by 9.8%. Although the training was only carried out over eight sessions, it caused similar increases as 14 training sessions over 6 weeks with threshold intensity (Chmura and Nazar, 2010). As such, the higher intensity (but not significantly above the ANT) specific HIIT used in the current study appears to be more time efficient.

Sperlich et al. (2011) found a 6.5% VO_2_ increase in young soccer players during a 5-week HIIT program, which is similar to VO_2_ found in our study. Therefore, it follows that the training comprising three exercises carried out between ANT and PFT, each of which generates longer duration, increased intensity, and greater stimulus variability, is a practical proposal that effectively increases the exercise capacity of soccer players.

These findings may also suggest that the specific HIIT proposed herein affects cognitive aspects. Indeed, PFT defining the highest CNS efficiency (shortest reaction time, best anticipation, perception, and optimal decision making) also shifted toward higher loads during this training (Chmura and Nazar, 2010), with significant increases in VO_2_ (5.9%), running speed (9.0%), and HR (0.9%). Such progression of PFT toward higher loads means, in practice, that the athlete can make optimal decisions at higher running speed and higher fatigue tolerance (Konefał et al., 2022). Given the very high intensity in modern soccer (Andrzejewski et al., 2018; Chmura et al., 2018; Konefał et al., 2019), such an effect is extremely desirable, as it allows players to perform actions efficiently at extremely intense moments of a match.

It makes sense for HIIT to include SSGs since the players would also improve technical and tactical elements and their decision-making processes, which are required for playing soccer (Davids et al., 2013; Trecroci et al., 2020) Therefore, the next aim was to determine if participants would exceed the ANT and achieve PFT intensity during four-versus-four matches on a 25 m × 35 m pitch that reflect the game conditions required in the specific HIIT proposed. The parameter frequently used in coaching practice to determine the effort intensity during the game is HR (Brandes et al., 2012; Aşçı, 2016; Konefał et al., 2023), which can be measured with precision and accuracy using available measurement technologies (Beenham et al., 2017; Gómez-Carmona et al., 2020; Pop et al., 2022). In this study, soccer players achieved HR max values that exceeded the intensity found at the ANT five-fold during six 3-min matches, even though they did not reach such intensity at the PFT during any of the games. However, it is worth noting that the HR max values were slightly lower than this intensity in the first two games.

It appears that the format used (four versus four) generated too little intensity in relation to the PFT. Therefore, the pitch size should be modified to slightly increase the game intensity (Aguiar et al., 2012), and the repetitions decreased to five to achieve the desired effects. Indeed, the analysis of kinematic parameters demonstrated a requirement of six consecutive games for the players to maintain their TDC, V max, and Acc levels. Nonetheless, values for V mean and Dec indicated a need to reduce the number of games to five since these parameters significantly decreased in the final game (Konefał et al., 2023).

The strength of this study is to find an appropriate exercise program that effectively increases the studied physiological variables in a short period of time while boosting performance. A high level of exercise capacity will allow coaches to concentrate more in training and on bringing out the technical and tactical potential of players. The authors are fully aware of various factors that could have influenced the results of the presented analyses. Indeed, experimenting on young players who are still developing their biological and psychomotor potential is a limitation of the study. A small sample size is another limitation. Therefore, these results should be treated with caution. In order to better understand the relationship between the specific HIIT proposed and the game format used (four versus four), further research should take into account various pitch size modifications (Hill-Haas et al., 2011; Arslan et al., 2017). Furthermore, it would be advisable to continue research on the relationship between ANT and PFT in the context of new training exercises (with and without the ball) that helps in developing both physical performance and decision-making, while accounting for different age groups and fitness levels.

## Conclusion

It was found that 4 weeks of specific HIIT significantly increased VO_2_ and running speed at the ANT and PFT, as well as HR at the ANT. These findings demonstrated that both thresholds shifted toward higher loads, which may be the result of adaptation to the applied training loads.

The participants exceeded ANT intensity but did not reach PFT intensity during four-versus-four soccer matches on a 25 m × 35 m pitch. However, they did approach PFT intensity during the first two games. Therefore, it would be advisable to modify the dimensions of the pitch in order to increase the intensity of the game. In addition, the analysis of kinematic parameters suggested that using five games would be more effective.

### Practical applications

Bringing out the full potential of players during the starting effort is invariably one of the most important goals of coaches, strength and conditioning coaches, and training staff. The proposed HIIT comprising three exercises carried out between ANT and PFT, each generating greater intensity, greater variability of the stimulus, and longer duration, is a practical proposal that effectively increases the exercise capacity of soccer players.

## Data Availability

The original contributions presented in the study are included in the article/supplementary material; further inquiries can be directed to the corresponding author.
